# Classification of brain disease in magnetic resonance images using two-stage local feature fusion

**DOI:** 10.1371/journal.pone.0171749

**Published:** 2017-02-16

**Authors:** Tao Li, Wu Li, Yehui Yang, Wensheng Zhang

**Affiliations:** 1 Institute of Automation, Chinese Academy of Sciences, Beijing, China; 2 University of Chinese Academy of Sciences, Beijing, China; National University of Defense Technology College of Mechatronic Engineering and Automation, CHINA

## Abstract

**Background:**

Many classification methods have been proposed based on magnetic resonance images. Most methods rely on measures such as volume, the cerebral cortical thickness and grey matter density. These measures are susceptible to the performance of registration and limited in representation of anatomical structure. This paper proposes a two-stage local feature fusion method, in which deformable registration is not desired and anatomical information is represented from moderate scale.

**Methods:**

Keypoints are firstly extracted from scale-space to represent anatomical structure. Then, two kinds of local features are calculated around the keypoints, one for correspondence and the other for representation. Scores are assigned for keypoints to quantify their effect in classification. The sum of scores for all effective keypoints is used to determine which group the test subject belongs to.

**Results:**

We apply this method to magnetic resonance images of Alzheimer's disease and Parkinson's disease. The advantage of local feature in correspondence and representation contributes to the final classification. With the help of local feature (Scale Invariant Feature Transform, SIFT) in correspondence, the performance becomes better. Local feature (Histogram of Oriented Gradient, HOG) extracted from 16×16 cell block obtains better results compared with 4×4 and 8×8 cell block.

**Discussion:**

This paper presents a method which combines the effect of SIFT descriptor in correspondence and the representation ability of HOG descriptor in anatomical structure. This method has the potential in distinguishing patients with brain disease from controls.

## Introduction

Magnetic resonance imaging (MRI) is a powerful technique which provides rich information about anatomical structure [1]. As a non-invasive medical imaging technique, MR images have been widely used in neuroscience and brain disease research [2]. Growing interest has been focused on the accurate classification of brain disease using MR images.

Many MRI measurements have been used as features and combined with classification methods to aid the diagnosis of brain disease. The traditional measures extracted from the structural MR images include: 1) Voxel-wise density [3–16]; 2) Cortical thickness [17–22]; 3) Volume [17,19,23–31]; 4) Deformation information [32,33]. However, there are several constraints in such MRI measures: 1) A fundamental assumption underlying these statistical measures is that inter-subject registration is capable of achieving one-to-one correspondence [35–37]. As the knowledge of correspondence is ambiguous or non-existent, this assumption may be potentially unrealistic [37,38]. 2) To extract these measures precisely, the brains require nonlinear alignments to a template. For the lack of ground truth, it's hard to evaluate the performance of inter-subject registration [39]. There is the risk of misalignment or over-alignment, and the differences caused by disease may be removed in the registration process [37,40]. 3) The cortical thickness and volume information are usually averaged from some predefined region of interest (ROI). These ROIs rely on manual or semi-automatic segmentation, which is time consuming and prone to errors [36].

To overcome the above limitations, many researches attempt to represent MR images using local features instead of MRI measures. Local features calculated independently from individual subject images help to identify the same anatomical structure among different subjects. Local features are combined with feature-based morphometry (FBM) and bag of words (BOW) to represent MR images in some recent studies. In FBM, the anatomical characteristics are represented by Scale Invariant Feature Transform (SIFT) features, and group-related anatomical differences are expressed in terms of feature/group co-occurrence statistics [37]. Afterwards, many extensions of FBM are proposed. Such as Mwangi et al. [12] propose to combine FBM with voxel-based morphometry (VBM), and obtain a good classification results. Chen et al. [40] propose a novel MR analysis method based on FBM and support vector machine (SVM). BOW is originally applied in document categorization, and is gradually used to analyze MR images recently. The basic idea of this method is to represent the image visual content as a probability distribution (histogram) of local features (visual words) and collect a knowledge based from a set of images previously labeled [41]. Local features such as SIFT features [42], Laguerre Circular Harmonic Functions coefficients [35,43] are combined with BOW in the classification of brain diseases.

However, there still have several constraints in the aforementioned FBM-based and BoW-based algorithms:

1. The location information of local features is ignored in BoW, thus the local features from different anatomical structure are substituted by the same visual word in the bag. The effect of local feature in correspondence would be weakened.
2. In FBM, some significant features come from few subjects. In practical, as the number of sample is limited, some significant features may be unrepresentative in a larger dataset.
3. In FBM, the SIFT features whose occurrence frequency are close in different groups provide little information for the final classification. As SIFT features are invariant to rotation and scale, two points which are similar with each other in SIFT descriptors may be different in the same scale.

To address the above constraints, in this paper, we aim to integrate the advantage of SIFT features in correspondence and the advantage of Histogram of Oriented Gradient (HOG) features in representation. Thus we propose a two-stage local feature fusion method. In correspondence stage, we extract keypoints and SIFT descriptor from scale-space in MR images. SIFT descriptors are used to correspond same anatomical structure among different subjects. The representative keypoints which exist in most of sample are reserved for they are more likely to appear in the test subjects. In representation stage, HOG descriptors will be calculated to demonstrate the local character around the representative keypoints from the same scale. The difference in HOG descriptors between two groups is used to weight the effect of the representative keypoints. SVM classifier is constructed for a representative keypoint in terms of HOG descriptors. For a new subject to be classified, we firstly identify matches of the representative keypoints, and then assign scores for the matches based on trained SVM classifier. The final classification result depends on the total score.

The contributions of this paper are as follows: 1) A two-stage local feature fusion method is proposed, in which SIFT features are used for correspondence and HOG features are used to character anatomical structure. 2) To reduce the calculation complexity and construct a standard keypoint set, the template brain is used as reference. 3) A quantification of representative effect of keypoints is adopted.

The rest of this paper is organized as follows. Firstly, the whole framework of classification via two-stage local feature fusion is introduced in Section 2. Then the experiments and results are presented in Section 3. Finally, the discussion and conclusion of this paper are followed in Section 4.

## Method

**Fig 1:**
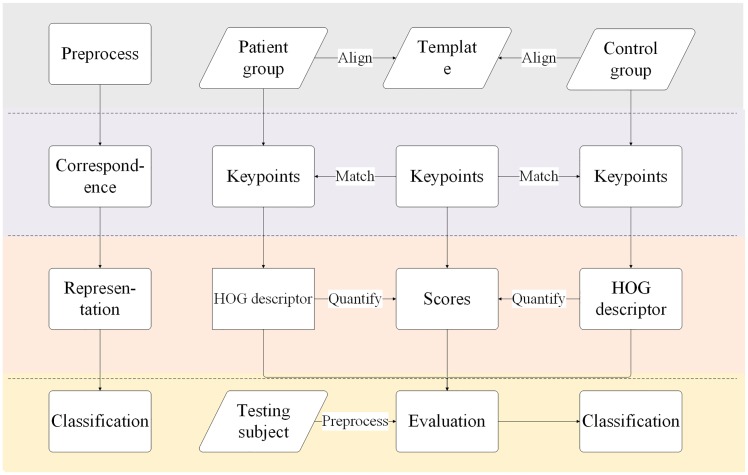
Flow diagram of proposed approach. The overall process contains the following steps: (1) the training MR images are aligned to template brain; (2) keypoints are extracted for every subject and matched among different subjects; (3) HOG descriptors are calculated for keypoints, and differences in terms of HOG is quantified. The effect of keypoint in classification is represented through assigning scores for them. (4) based on keypoints and their scores, testing subject is classified.

The overall flow of proposed framework is illustrated in Fig 1. This framework can be broke up into four steps, which is summarized as follows: preprocessing, correspondence, representation, and classification.

### Preprocessing

The proposed method in this study is evaluated on public datasets in Open Access Series of Imaging Studies (OASIS) [44] for Alzheimer's disease (AD) and Parkinson's Progression Markers Initiative (PPMI) [45] for Parkinson's disease (PD).

#### OASIS dataset

OASIS contains 98 normal control (NC) subjects and 99 probable AD subjects aged 60–96. For the later comparison, these subjects are subdivided into two subsets according to their ages and dementia statuses measured by Clinical Dementia Rating (CDR):

1. AD-86: 86 subjects aged 60–80 years are chosen, including 20 patients with mild AD (CDR = 1) and 66 healthy subjects (CDR = 0) [40];
2. AD-126: 126 subjects aged 60–96 years are chosen, including 28 patients with mild AD (CDR = 1) and 98 healthy subjects (CDR = 0) [40].

Scans in OASIS subjects are first averaged and gain-field corrected in advance to improve signal/noise ratio, and then registered to Talairach space via affine transform and the skull are masked out. Marcus et al [44] present the detailed description of the preprocessing steps for this dataset. These subjects are subdivided into two subsets according to their ages and dementia statuses measured by Clinical Dementia Rating.

#### PPMI dataset

The PPMI cohort comprises more than 400 Parkinson's disease (PD) subjects and more than 200 healthy subjects acquired from 21 clinical sites with different scanner parameters [45]. We use T1-weighted MP-RAGE images whose slice thickness is 1.0mm and acquisition plane is sagittal. We found 67 NC subjects and 145 PD subjects. These subsets will be used to evaluate the usability of this method. These subjects are subdivided into two subsets according to their ages and depression statuses measured by Geriatric Depression Scale (GDS):

1. PD-46: 46 subjects aged 50–65 years are chosen, including 20 healthy subjects (GDS<4) and 26 PD subjects (GDS>4).
2. PD-212: 212 subjects aged 30–85 years are chosen, including 67 healthy subjects and 145 PD subjects.

### Correspondence

We extract maxima and minima of scale-normalized Laplacian of Gaussian [46]. These extremal points are considered as keypoints in this paper, which stand for anatomical structures. SIFT features are widely used in registration for its invariance to image scale and rotation. In this paper, we use SIFT features to correspond same anatomical structure (keypoint) among different subjects.

In correspondence part, firstly, we extract keypoints from scale-space and calculate SIFT descriptors for them in train subjects and template brain ICBM_152. Secondly, for a keypoint in template brain, we locate matches in training subjects. The last but not the least, keypoints which find matching points in few subjects are filtered out.

#### Extracting keypoints and SIFT descriptors

**Fig 2:**
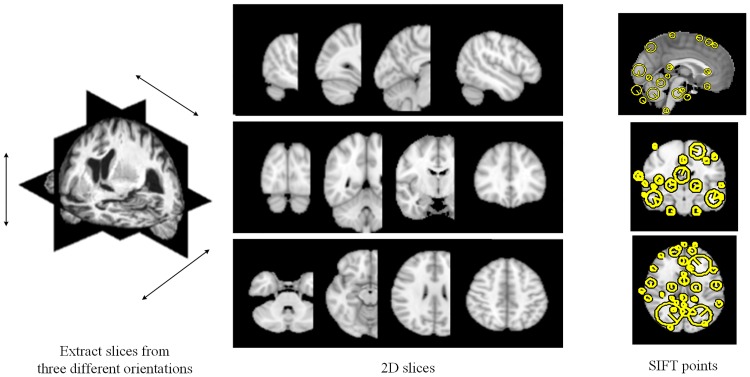
3D volume is represented by 2D features. The brain is sliced along 3 orientations to obtain 2D images, and SIFT features are extracted from 2D images.

After transforming 3-D brain images to 2-D slices as shown in Fig 2, package vlFeat [47] is used to extract keypoints and SIFT descriptors from these slices. The SIFT descriptor is described by 128 numbers which characterize the image appearance around the keypoint in more detail. A keypoint corresponds to a slice orientation (coronal, axial or sagittal), a slice order of the 2-D slice from which the keypoint is extracted, an orientation, a central location. We extract keypoints and SIFT descriptors for every subject in patient group, normal control group and the template brain ICBM_152. To be noted, the appearance matrix is stretched into vector, and the vector is normalized to unit length.

Here, an individual feature is denoted as *f*_*i*_ = {*d*_*i*_, *l*_*i*_, *x*_*i*_, *o*_*i*_, *a*_*i*_} where *d*_*i*_ and *l*_*i*_ are the slice orientation and slice order of the 2-D slice respectively, and *x*_*i*_ is the center location of keypoint, and *a*_*i*_ is a vector of image measurements representing the image appearance around keypoint. Let *F*_*p*,*m*_(*m* ∈ [1, *M*]), *F*_*c*,*n*_(*n* ∈ [1, *N*]) and *F*_*t*_ represent all keypoints extracted from the *m*th subject in patient group, the *n*th subject in control group and the template brain ICBM_152. Where *M* is the total number of patient subjects, and *N* is the total number of nomal control subjects. To illustrate this method more clearly, keypoint is expressed in different ways. In this paper, the keypoint in template brain is still called "keypoint", and the keypoint in training and testing subjects is called "candidate point". For a keypoint in template, the correspondence found in training and testing subjects is called "matching point".

#### Locating matching points

To locate matching point in training subjects, a common method is clustering. However, two or more candidate points of one subject may be assigned into one cluster due to noise or clustering algorithm. In this work, we propose a concise method to accomplish this goal via template brain. We assume that the candidate points which appear in training and testing subjects can all be covered by keypoints extracted from template brain ICBM_152.

The matching algorithm is performed between one image in template brain and the image in training subjects which shares the same slice orientation and slice order. For example, suppose *f*_*i*_ is a keypoint extracted from the brain template, and we plan to locate the matching point in the *m*th subject of patient group. The matching algorithm can be subdivided into three steps:

Firstly, from *F*_*p*,*m*_, we retrieve the candidate points which share the same slice orientation and slice order with *f*_*i*_,

$$F_{temp}=\{f_{j}|f_{j}\in F_{p,m}\wedge d_{j}=d_{i}\wedge l_{j}=l_{i}\}$$ (1)

Secondly, we calculate the Euclidean distance for the invariant descriptor vector between *f*_*i*_ and keypoint *f*_*j*_ in *F*_*temp*_.

Thirdly, we compare the Euclidean distance of the closest neighbor to that of the second-closest neighbor. If this distance ratio is smaller than *ε*_*match*_, we believe that we find the matching point in the *m*th subject of patient group.

The idea behind this matching algorithm is that correct matching point should have the closest neighbor significantly closer than the closest incorrect matching point to achieve reliable matching [48]. This matching algorithm is based on Lowe's work [48]. Specially, if there is just one candidate point in *F*_*temp*_, we don't believe that we have found the correct matching point.

#### Discarding unrepresentative keypoints

For a keypoint *f*_*i*_ in template brain, we identify the matching points in patient group and control group. For keypoint *f*_*i*_, let *S*_*i*,*p*_ and *S*_*i*,*c*_ be the set of matching keypoints in patient group and normal control group respectively. In this paper, we aim to find the representative keypoints (anatomical structure) which appear in most of subjects. Therefore, the keypoints which find matching points in a fraction of subjects are not what we pursue. Specifically, if |*S*_*i*,*p*_|< *ε*_*rate*_ × *M* or |*S*_*i*,*c*_|< *ε*_*rate*_ × *N*, we consider *f*_*i*_ a unrepresentative keypoint, otherwise we believe *f*_*i*_ is a effective keypoint. Where |⋅| is the cardinal number of a set.

After discarding unrepresentative keypoints, the total number of keypoints in template brain is reduced to *K*. Namely, for every effective keypoint *f*_*i*_ in template brain, we have a patient matching set *S*_*i*,*p*_ and normal control matching set *S*_*i*,*c*_.

### Representation

In representation part, firstly, histogram of oriented gradients (HOG) descriptors is extracted for matching points. Then, the effect of keypoint in classification is quantified based on the difference of two groups in HOG descriptor. Finally, a SVM classifier is constructed for every keypoint.

#### HOG descriptors

**Fig 3:**
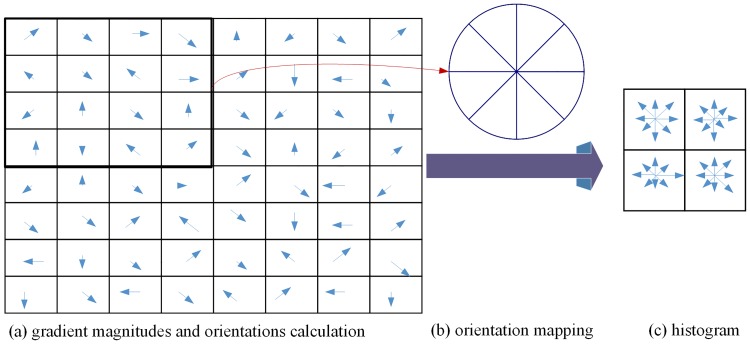
Illustration of the extraction of local feature (HOG). Gradient magnitudes and orientations are sampled, and then accumulated into orientation histogram with 8 channels. (a) Cell block. (b) 8 histogram channels. (c) Oriented gradients matrix.

SIFT feature is invariant to scale, which indicates that the appearance matrix of a keypoint may be calculated in different scales. To demonstrate the differences in morphology of the same anatomical structure between different groups better, we extract local features in the same scale (the original image). A local feature similar with HOG descriptor is used to depict the gradient character of a block around the keypoint. For a matching point *f*_*i*_ = {*d*_*i*_, *l*_*i*_, *x*_*i*_, *o*_*i*_, *a*_*i*_}, the extraction of local feature can be described as:

1. To make the local feature (HOG) be rotationally invariant, the image from which keypoint *f*_*i*_ is extracted should be rotated relative to the dominant orientation *o*_*i*_ in advance.
2. The image gradient magnitudes and orientations are sampled in a 16×16 cell block shown in Fig 3(a) around the keypoint location *x*_*i*_.
3. Gradient magnitudes are accumulated into orientation histograms summarizing the contents over 4×4 subregions with 8 histogram channels shown in Fig 3(b) and 3(c).
4. The description matrix is converted to a local feature (HOG) vector, and the vector is then normalized to unit length.

The final local feature (HOG) vector is a geometric description of keypoint. Similar calculation is done for the matching points in *S*_*i*,*p*_ and *S*_*i*,*c*_. The local features extracted for *S*_*i*,*p*_ and *S*_*i*,*c*_ are denoted by *Q*_*i*,*p*_ and *Q*_*i*,*c*_. The dimensions of matrix *Q*_*i*,*p*_ are *H* × |*S*_*i*,*p*_|, and the dimensions of matrix *Q*_*i*,*c*_ are *H* × |*S*_*i*,*c*_|. Where |⋅| is the cardinal number of a set, and *H* is the length of a HOG feature vector.

#### Quantification

In this section, we will evaluate the difference in geometry of this anatomical structure between patient group and normal control group. RV coefficient [49] will be used to implement this difference measure.

For keypoint *f*_*i*_, the local features (HOG) in patient group and normal control are *Q*_*i*,*p*_ and *Q*_*i*,*c*_ respectively. The RV coefficient for these two set can be described as:

$$RV(Q_{i,p},Q_{i,c})=\frac{tr(Q_{i,p}Q_{i,p}{}^{T}Q_{i,c}Q_{i,c}{}^{T})}{tr{\left(Q_{i,p}Q_{i,p}{}^{T}Q_{i,p}Q_{i,p}{}^{T}\right)}^{\frac{1}{2}}\times tr{\left(Q_{i,c}Q_{i,c}{}^{T}Q_{i,c}Q_{i,c}{}^{T}\right)}^{\frac{1}{2}}}$$ (2)

Where *tr*(.) is the trace operator of square matrix, *Q*^*T*^ is the transpose of matrix *Q*.

The value of RV coefficient ranges from 0 to 1. If RV coefficient is 0, the two sets are independent, which means there is no correlation or similarity between the two data sets [50]. If RV coefficient is 1, the eigen components of data set *Q*_*i*,*p*_ can be derived from *Q*_*i*,*c*_ through a homothetic transformation, which means that there exists a rotation matrix *H* and a scaling factor *c* such that *cQ*_*ip*_*H* = *Q*_*ic*_. Namely, larger RV coefficient means more similarity.

The effect of keypoint *f*_*i*_ in classification is quantified in terms of the differences in local feature (HOG). As *RV*(*Q*_*i*,*p*_, *Q*_*i*,*c*_) reflects the similarity between the two data sets *Q*_*i*,*p*_ and *Q*_*i*,*c*_. We use 1−*RV*(*Q*_*i*,*p*_, *Q*_*i*,*c*_) to represent the degree of difference between control group and patient group at keypoint *f*_*i*_. A keypoint is scored according to following expression:

$$f_{i}score=(1-RV(Q_{i,p},Q_{i,c}))$$ (3)

#### SVM classifier

For a keypoint *f*_*i*_(*i* ∈ [1, *K*]) in template brain, the local features (HOG) in patient group and normal control are *Q*_*i*,*p*_ and *Q*_*i*,*c*_ respectively. We train a SVM classifier for this keypoint to identify a new local feature is healthy feature or patient feature. The input samples to the SVM are *Q*_*i*,*p*_ and *Q*_*i*,*c*_. The class labels are -1 and 1 if they are from healthy group and patient group respectively. A 2-class SVM implementation from libSVM [51] is used. As the number of feature is in the same order as the number of samples, linear SVM is chosen to classification. A linear SVM classifier is less prone to overfitting than a non-linear one.

To be noted, *Q*_*i*,*p*_ and *Q*_*i*,*c*_ are imbalanced, and a simple default strategy of guessing the majority class would give a nice classification results. To make the classifier more suitable, we use an over-sampling approach in which the minority class is over-sampled by creating "synthetic" examples [52]. SMOTE arithmetic is used in this paper.

### Classification

For a testing subject to be classified, candidate points and SIFT descriptors *F*_*test*_ are first extracted. Based on the effective keypoint *f*_*i*_(*i* ∈ [1, *K*]) in brain template, the keypoint score *f*_*i*_*score* and corresponding SVM classifier, we classify the testing brain in following steps:

1. Locating matching point. For a keypoint *f*_*i*_(*i* ∈ [1, *K*]) in template brain, we identify whether this anatomical structure exists in *F*_*test*_. The matching algorithm has been presented in Correspondence.
2. Extracting local features. If there exists a matching point in *F*_*test*_ for *f*_*i*_(*i* ∈ [1, *K*]), we extract HOG descriptor *θ*_*i*_ at the central location based on method presented in Representation.
3. Assigning scores. For local feature *θ*_*i*_, we label it based on the trained SVM classifier:
  $$LS(\theta_{i})=\{\begin{array}{ccc} f_{i}score & & \begin{array}{cc} patient & feature \end{array}\\ -1\times f_{i}score & & \begin{array}{cc} healthy & feature \end{array} \end{array}$$ (4)
4. Classifying new subject. The final classification result depends on the sum of scores *LS*_*sum*_ = ∑_*i*_*LS*(*θ*_*i*_) of all matched keypoints. The brain is classified as patient brain if *LS*_*sum*_ is larger than threshold *ε*_*c*_ and is classified as normal control brain otherwise.
  $$classlabel==\{\begin{array}{cc} Patient & LS_{sum}>\varepsilon_{c}\\ Control & otherwise \end{array}$$ (5)

Ideally, the threshold is 0, however, the occurrence frequency of keypoints in patient group and control group is inconsistent. We obtain this threshold similar with Chen et al.' [40] method by classifying the training brains and then finding the threshold that minimize the difference between false negatives and false positives. Finally, we use this threshold to predict new brains.

### Parameter setting

There are several thresholds including matching threshold *ε*_*m*_, and RV threshold *ε*_*rv*_. Matching threshold *ε*_*m*_ is set as 0.8 which follows the suggestion of Lowe's paper [48]. Rate threshold *ε*_*rate*_ is set as 0.5. The size of the cell block also affects the final result. Different sizes of cell block are tested in the following evaluation.

### Ethical standards and patient consent

Datasets AD-86 and AD-126 are obtained from the OASIS database. Written informed consent is obtained from all subjects and the use of these subjects is approved by Washington University (http://www.oasis-brains.org/).

Datasets PD-46 and PD-212 are obtained from the PPMI database. PPMI database is funded by the Michael J. Fox Foundation for Parkinson’s Research and funding partners, including Abbvie, Avid, Biogen Idec, Br Bristol-Meyers Squibb, Covance, GE Health care, Genentech, GlaxoSmithKline, Lilly, Lundback, Merck, Meso Scale Discovery, Pfizer, Piramal, Roche, Servier, and UCB (www.ppmi-info.org/fundingpartners). Written informed consent is obtained from all subjects.

## Experiment and results

### Visualization of keypoints

**Fig 4:**
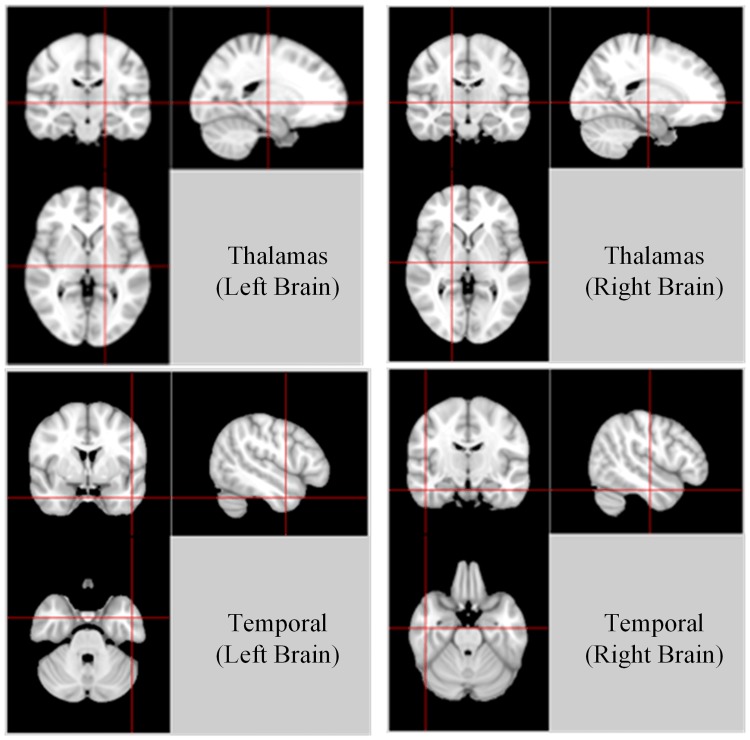
Illustration of some keypoints with a high score. Four keypoints are listed, which appear in the same anatomical structure of left brain and right brain with a high score.

FBM-based methods and BOW-based methods try to utilize the unbalance of SIFT features occurrence in different groups. However, our method tries to identify the keypoints which exist difference in anatomical characteristics from same image scale. To identify the discriminative brain regions, AAL atlas was used to map the keypoints. Examples of 4 keypoints for OASIS which appear in the same anatomical structure of left brain and right brain with a high score are shown in Fig 4.

For AD discrimination, the keypoints which have a high score are distributed mainly in thalamas, temporal lobe, calcarine and cingulum. A number of papers have reported these four regions [53–56]. Thalamas is responsible for the regulation of consciousness [57]. AD patients are usually loss of motivation and not managing self care [58]. Thalamas may be related with this symptom. The temporal lobe is very reasonable region with considerations of its important role in processing sensory input to derived meanings [59]. Temporal lobe is an import part for the appropriate retention of visual memory, language comprehension and emotion association, which is associate with the symptom (short-term memory loss, emotion swing) of AD [58,59]. Cingulum is very important to brain structure connectivity and information integration [60]. Damage in this area may result in many mental disorders.

For PD discrimination, the most significant keypoints are distributed mainly in thalamas, frontal lobe, occipital lobe which in accord with many papers [61–63]. Thalamas is responsible for the regulation of consciousness [57]. This is in consist with PD patients' symptoms such as sensory and emotional problems [64]. The frontal lobe is an very important part for voluntary movement which may cause the behavioral problems in PD patients [65].

### Evaluation and performance

We perform leave-one-out cross validation for all datasets. One subject is chosen as testing subject once and the remaining subjects are used for training. The accuracy, sensitivity and specificity of the classification are computed for all datasets.

#### Keypoints

Intuitively, we assume that the keypoints which present more difference between two groups contribute more to the final classification.

**Fig 5:**
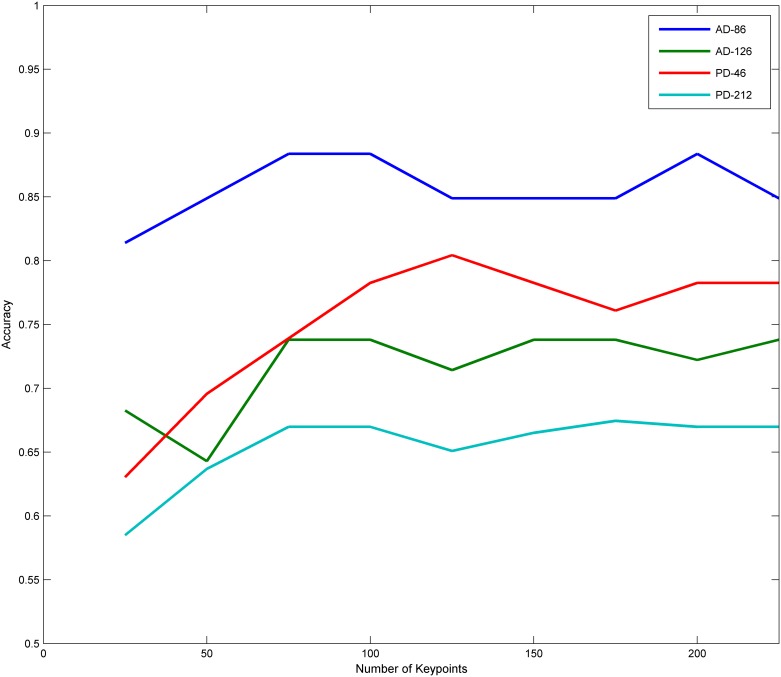
Classification accuracy with different numbers of keypoints. As the number of keypoints increases, the classification accuracy changes in AD-86, AD-126, PD-46 and PD-212.

In this section, we take the top 25, 50,…, 225 keypoints ranking according the difference of HOG descriptors between groups. The classification results for AD-86, AD-126, PD-46 and PD-212 are illustrated in Fig 5. For AD-86 and PD-46, as the number of keypoints increases, the classification accuracy increased at first and then decreased. For AD-126 and PD-212, the method obtains best performance when taking 75 or 100 keypoints. Then the performance swings a little. From the curve in Fig 5, we find that classification results in four groups with 100 keypoints are not bad. In this paper, we take the top 100 keypoints ranking according to the difference of HOG descriptors for the classification of AD-86 and AD-126.

To verify our assumption, we take another test. In this paper, we propose to quantify the effects of keypoints in terms of the difference in local feature (HOG). The simplest method to quantify keypoints is assigning same weight for all keypoints. This corresponding to setting *f*_*i*_*score =* 1. Two types of scoring method are compared in this paper.

$$\text{Type }1:f_{i}score=1$$ (6)

$$\text{Type }2:f_{i}score=(1-RV(Q_{i,p},Q_{i,c}))$$ (7)

The comparison is done in group AD-86 and AD-126 shown in Table 1. For AD-86, PD-46 and PD-212, there is an improvement in the classification accuracy when assigning weight for keypoints in terms of the difference of local feature (HOG). However, for AD-126, assigning weights for keypoints leads to a lower classification accuracy. This may be due to the effect of keypoints which have a low score. These keypoints affect the final results.

**Table 1:** Classification results for different score methods.

Type		Accuracy(%)	Sensitivity(%)	Specificity(%)
**AD-86**	Type 1	84.88	85.00	84.85
	Type 2	86.05	85.00	86.36
**AD-126**	Type 1	76.19	78.57	72.45
	Type 2	74.60	75.00	74.49
**PD-46**	Type 1	73.91	73.08	75.00
	Type 2	78.26	76.92	80.00
**PD-212**	Type 1	65.57	67.16	64.83
	Type 2	66.98	70.15	65.52

#### Cell block for local feature HOG

Local feature (HOG) is extracted from cell blocks. The size of cell blocks plays an important effect on the final classification. Three types of cell blocks are compared in this paper, and the performance is displayed in Table 2.

**Table 2:** Classification results for different cell block size.

Cell block size		Accuracy(%)	Sensitivity(%)	Specificity(%)
**AD-86**	4×4	75.58	75.00	75.76
	8×8	84.88	85.00	84.85
	16×16	86.05	85.00	86.36
**AD-126**	4×4	67.46	67.86	67.35
	8×8	69.05	67.86	69.39
	16×16	74.60	75.00	74.49
**PD-46**	4×4	69.57	69.23	70.00
	8×8	76.09	73.08	80.00
	16×16	78.26	76.92	80.00
**PD-212**	4×4	59.43	62.69	57.93
	8×8	65.09	65.67	64.83
	16×16	66.98	70.15	65.52

The results in Table 2 suggest that a bigger cell block leads to a better result in general. HOG descriptor extracted from a larger region may be more likely to demonstrate the detailed information around the keypoint. The results also suggest that the oriented gradients matrix in a small region would be hard to describe the detailed structural around the keypoint, and the difference between group would be deviated from the reality. In order to balance the speed and the accuracy, 16×16 cell blocks will be used in this paper.

#### Different local feature for representation

We try different types of local feature for representation and analyze which kind of local feature is suitable to demonstrate difference between healthy control group and patient group. Three kinds of local feature are compared in this paper: gray value (GV), gray-level co-occurrence matrix (GLCM) and HOG. The performance is displayed in Table 3. From the result, we find that classification with GLCM in AD-86 and AD-126 is very poor, this may due to that sulcus is widespread exists and GLCM is difficult to describe the difference. The classification results using GLCM and gray value are similar in PD-46 and PD-212. Though worse than HOG, gray value may be able to provide effective information for the final classification.

**Table 3:** Classification results for different local feature.

Representation		Accuracy(%)	Sensitivity(%)	Specificity(%)
**AD-86**	GV	81.40	80.00	81.82
	GLCM	76.74	75.00	77.27
	HOG	86.05	85.00	86.36
**AD-126**	GV	70.63	71.43	70.41
	GLCM	62.70	64.29	62.24
	HOG	74.60	75.00	74.49
**PD-46**	GV	71.74	69.23	75.00
	GLCM	73.91	73.08	75.00
	HOG	78.26	76.92	80.00
**PD-212**	GV	64.62	65.67	64.14
	GLCM	63.21	62.69	63.45
	HOG	66.98	70.15	65.52

#### Effect of local feature (SIFT) in Correspondence

To demonstrate the effect of local feature (SIFT) in correspondence, we compare the classification results with and without the use of SIFT. With the help of SIFT, we extract local feature (HOG) at the keypoints extracted from scale-space in training subjects. Without the use of SIFT, based on the central location of keypoints extracted from the brain template, we extract local feature (HOG) at the same position in training subjects. The comparison is done in four groups.

The performance of four datasets with and without the effect of local feature (SIFT) in correspondence is shown in Table 4. The results suggest that with the help of local feature (SIFT) in correspondence, the performance becomes much better. Keypoints extracted from scale-space help to locate the coordinate and SIFT features help to correspond among different subjects. This improvement supports the idea that local feature help to relieve the constraint in registration.

**Table 4:** Classification results with or without the use of SIFT features.

SIFT features		Accuracy(%)	Sensitivity(%)	Specificity(%)
**AD-86**	With	86.05	85.00	86.36
	Without	81.40	85.00	80.03
**AD-126**	With	74.60	75.00	74.49
	Without	71.43	67.86	72.45
**PD-46**	With	78.26	76.92	80.00
	Without	71.74	73.08	70.00
**PD-212**	With	66.98	70.15	65.52
	Without	66.04	64.18	66.90

#### Accuracy

For better comparison, we compare with methods which have been tested on the OASIS dataset. The subjects in this dataset have been preprocessed, avoiding the possible noise in preprocessing.

Toews et al. [37] propose a feature-based morphometry (FBM), in which group-related anatomical differences are expressed in terms of feature/group co-occurrence statistics. Daliri [42] proposes to combine BOW with SIFT features, followed by classification with SVM. Chen et al. [40] propose a MR analysis method based on FBM and SVM. Wang et al. [66] improve correspondences of localized patterns based on FBM. These four methods are all evaluated on AD-86 and AD-126. The comparison is summarized in Table 5. Equal Error Rate (EER) accuracy is used to evaluate the performance of classification method, so we follow this criterion. EER accuracy is calculated by first choosing a threshold so that the false positives rate is equal to false negative rate and then calculating the classification accuracy with the chosen threshold [37,40].

**Table 5:** Classification results in the literature: SIFT-based methods.

	AD-86	AD-126
Toews et al.' method	80	70
Daliri's method	86	78
Chen et al.' method	83	71
Wang et al.' method	80	79
Proposed	88	78

## Discussion

### Local features

There are many local features such as SURF [67], LBP [68], Extended LBP [69], WHGO [70], Multi-view feature [71] and so on, it's hard to select the best one. In this paper, we mainly demonstrate one method which combines the advantage of local feature in correspondence among different subjects and representation of anatomical structure. In this paper, we choose SIFT feature for correspondence and choose HOG feature for representation of anatomical structure.

SIFT features are widely used in registration to compensate the deformation of anatomical structures and achieve effective alignment among different subjects [72]. We choose SIFT feature for correspondence mainly due to its robustness and invariance for rotation and scale. Due to individual difference, same structure in different subjects may have great differences in the same scale. As SIFT features are invariant to scale, same structure can be robustly corresponded among different subjects. The performance of AD-86, AD-126, PD-46 and PD-212 with and without the effect of local feature (SIFT) in correspondence is displayed in Table 4. The result shows that SIFT features may help to achieve more precise correspondence among subjects and help to obtain better classification results.

The other local feature we use in this paper is designed to discriminate different structural pattern. Three kinds of local feature (GV, GLCM and HOG) are used to depict the texture around the keypoints and search difference between different groups. The performance in Table 3 shows that HOG outperforms GV and GV outperforms GLCM. GLCM perform very poor which beyond our expectation. We find that the numbers in the corners of GLCM are usually much larger than the numbers in the center of GLCM. This may be due to the structure of sulcus. Sulcus is so widespread that GLCM is difficult to reflect differences between groups. The results don't mean that HOG is the most suitable local feature for representation. The results reflect that gradient information outperforms gray value adjacency information and gray value. This may result from the special structural pattern of brain sulcus.

HOG features are finally selected to demonstrate the detailed gradient information of a cell block. The cell block in which we extract HOG descriptors plays an important role in classification as shown in Table 2. Measurements extracted from a small scale are limited in representation, leading to the bad performance in 4×4 cell block. Our results show that 16×16 outperforms 4×4 and 8×8. Measurements from moderate scale can be effectively useful for the representation of anatomical characteristics.

### Keypoints

The keypoints extracted from MR images are used to represent the anatomical structure. The representation of 3-D images is greatly simplified via keypoints and template brain. Moreover, the keypoints extracted from maxima and minima of scale-normalized Laplacian of Gaussian produce the most stable local image features compared to a range of other points [46]. Some studies simplify the representation of 3-D images by t-test. For example, Hinrichs et al. [34] consider each voxel's intensity value as feature, and t-test is performed to rank the features by resulting p-values. The top ranked features are seen as a simplification of original 3-D image. This simplification is based on the assumption that one-to-one correspondence can be achieved among subjects precisely. As mentioned before, this assumption is ambiguous or non-existent. Besides, the significant region appears in the form of clusters, which result in redundancy. In this paper, we simplify MR images via keypoints. On one hand, keypoints can provide precise correspondence. On the other hand, simplification through keypoints is very concise, and a 3-D MR image can be represented by only five thousands of voxels.

### Scoring methods for keypoint

In this paper, RV coefficient is used to measure the similarity between two sets of local features. We assume that the keypoints which have little difference in terms of HOG descriptors play little effect on the final classification. The results in Fig 5 and Table 1 suggest this assumption is reasonable. When the RV coefficient is high, we assume that there is no difference between two local feature groups. The results using this strategy shown in Table 1 perform well in terms of accuracy.

### Limitation and future work

We use a 2-D SIFT algorithm mainly due to its robustness and feasibility, as many 3D SIFT implementations couldn't achieve the full orientation invariance respect to 3 degree of rotation freedom such as the 3DSIFT Matlab package [73]. Besides, for the limitation of computing capability in our lab, the serial images are down-sampled once to reduce the computation load. From the results testing on AD-86 and AD-126, we find that RV coefficient is sensitive to the number of sample. A more suitable quantification method should be studied for the difference of local features around keypoints in the future work.

## Conclusion

We propose a novel framework for the diagnosis of brain diseases from MR images based on local features. Two kinds of local features are embedded in this frame, one for correspondence and the other for representation. Keypoints are extracted to represent anatomical structure. Scores are assigned for keypoints to quantify their effect in classification. The sum of scores for all keypoints is used to determine which group the test subject belongs to.

The proposed framework is evaluated on public dataset OASIS and PPMI. As shown in Table 4, the proposed method outperforms four SIFT-based methods in terms of EER accuracy more or less. The results suggest that the proposed method can be potentially a practical means to represent anatomical characteristics and aid to diagnose brain diseases from normal controls.
